# Effect of apo-lactoferrin on leukotoxin and outer membrane vesicles of *Mannheimia haemolytica* A2

**DOI:** 10.1186/s13567-020-00759-z

**Published:** 2020-03-05

**Authors:** Christian Avalos-Gómez, Magda Reyes-López, Gerardo Ramírez-Rico, Efrén Díaz-Aparicio, Edgar Zenteno, Cynthia González-Ruiz, Mireya de la Garza

**Affiliations:** 1 Facultad de Medicina Veterinaria y Zootecnia, Universidad Nacional Autónoma de México (UNAM), 04510 Coyoacán, CdMx Mexico; 2 Departamento de Biología Celular, Centro de Investigación y de Estudios Avanzados del Instituto Politécnico Nacional (CINVESTAV-IPN), Ave. Instituto Politécnico Nacional 2508, Zacatenco, 07360 CdMx, Mexico; 3 Facultad de Estudios Superiores Cuautitlán, Universidad Nacional Autónoma de México (UNAM), 54714 Cuautitlán Izcalli, Estado de México Mexico; 4 Centro Nacional de Investigación Disciplinaria en Salud animal e inocuidad, Instituto Nacional de Investigaciones Forestales, Agrícolas y Pecuarias (INIFAP), 05110 Cuajimalpa, CdMx Mexico; 5 Departamento de Bioquímica, Facultad de Medicina, Universidad Nacional Autónoma de México (UNAM), 04510 Coyoacán, CdMx Mexico

## Abstract

*Mannheimia haemolytica* serotype A2 is the principal cause of pneumonic mannheimiosis in ovine and caprine livestock; this disease is a consequence of immune suppression caused by stress and associated viruses and is responsible for significant economic losses in farm production worldwide. Gram-negative bacteria such as *M. haemolytica* produce outer membrane (OM)-derived spherical structures named outer membrane vesicles (OMVs) that contain leukotoxin and other biologically active virulence factors. In the present study, the relationship between *M. haemolytica* A2 and bovine lactoferrin (BLf) was studied. BLf is an 80 kDa glycoprotein that possesses bacteriostatic and bactericidal properties and is part of the mammalian innate immune system. Apo-BLf (iron-free) showed a bactericidal effect against *M. haemolytica* A2, with an observed minimal inhibitory concentration (MIC) of 16 µM. Sublethal doses (2–8 µM) of apo-BLf increased the release of OMVs, which were quantified by flow cytometry. Apo-BLf modified the normal structure of the OM and OMVs, as observed through transmission electron microscopy. Apo-BLf also induced lipopolysaccharide (LPS) release from bacteria, disrupting OM permeability and functionality, as measured by silver staining and SDS and polymyxin B cell permeability assays. Western blot results showed that apo-BLf increased the secretion of leukotoxin in *M. haemolytica* A2 culture supernatants, possibly through its iron-chelating activity. In contrast, holo-BLf (with iron) did not have this effect, possibly due to differences in the tertiary structure between these proteins. In summary, apo-BLf affected the levels of several *M. haemolytica* virulence factors and could be evaluated for use in animals as an adjuvant in the treatment of ovine mannheimiosis.

## Introduction

*Mannheimia haemolytica* is a normal inhabitant of the nasal cavity and tonsil crypts of healthy ruminants. However, when animals suffer from shipping stress and/or an acute infection by *Mycoplasma bovis* (in cattle) or viruses (e.g., parainfluenza-3, adenovirus, and respiratory syncytial virus), they become immunocompromised. This condition leads to the rapid proliferation of *M. haemolytica*, which subsequently reaches the lungs and infects the alveolar epithelium. *M. haemolytica* serotype A2 causes acute pneumonic mannheimiosis in lambs, sheep and goats. Several studies have demonstrated the importance of mannheimiosis as a cause of mortality in these livestock species, as well as the negative effects on weight gain and a low efficiency in feed conversion in chronically affected sheep [1, 2]. In the USA, the occurrence of this disease in cattle (bovine respiratory disease, BRD) caused by serotype A1 results in productivity losses of 23.60 USD per sick calf and is a major cause of economic losses to farms [3]. The BRD complex accounts for approximately 70 to 80% of the morbidity and 40 to 50% mortality of cattle [4]. However, corresponding data with respect to serotype A2 is currently unavailable.

*Mannheimia haemolytica* A2 produces several virulence factors that together lead to acute fibrinous pleuropneumonia in sheep. The most important of these virulence factors is the leukotoxin (Lkt), a member of the RTX family of toxins from Gram-negative bacteria that is primarily secreted during the bacterial logarithmic growth phase. Lkt is a 104-kDa, thermolabile soluble protein that is toxic to ruminant macrophages, leukocytes and erythrocytes. Interestingly, the N-terminal region of Lkt has been shown to interact through nonspecific (electrostatic) contacts and through a specific protein receptor (β-integrin) of the target cells to mediate pore formation and lysis [5–10].

Gram-negative bacteria typically produce outer membrane (OM)-derived spherical structures named outer-membrane vesicles (OMVs) harboring biologically active proteins and other virulence factors that have various functions. The release of OMVs increases when bacteria are subjected to stress conditions, such as the addition of gentamicin [11, 12]. OMVs are 50–250 nm in diameter, and as they originate from the OM, they possess LPS, phospholipids and OM proteins (OMPs). In addition, OMVs harbor periplasmic components, such as enzymes and DNA fragments. The results of previous studies suggest that the OMV-mediated transport of virulence factors has major advantages compared with their conventional transport, since the molecules are packed into a structure that protects them (i.e., the membrane forming the vesicle) [13]. Our group has demonstrated that *M. haemolytica* A2 contains several immunogenic OMPs inside the OMVs, among which three proteins of 45, 54 and 60 kDa have been shown to react with the sera of sick sheep. In addition, the presence of Lkt, LPS, and a 23-kbp DNA fragment [11]. Cysteine- and metallo-proteases have been detected in OMVs based on zymography assays [14].

In recent years, the testing of *M. haemolytica* A2 isolates from animals with mannheimiosis has revealed increasing proportions of antimicrobial resistance [15–17]. In addition, due to the lack of a vaccine with 100% efficacy, new strategies to reduce the presence of this disease in farms has emerged. Lactoferrin (Lf) is a glycoprotein that belongs to the mammalian innate immune system possessing bactericidal and bacteriostatic effects and exhibiting immune regulatory functions. Lf is an 80 kDa protein with high homology among mammalian species [18] that is produced by glandular epithelial cells and secreted to the mucosae and by neutrophils at infection sites in all mammals. Lf is a non-haem iron-binding protein that is a member of the transferrin family, which includes serum transferrin and other proteins. It is now accepted that apo-Lf plays a direct antimicrobial role in secretions and epithelial surfaces by limiting the proliferation and adhesion of microbes and by frequently killing them. The bacteriostatic effect of apo-Lf has been attributed to its ability to capture Fe^3+^ ions and limit their use by pathogenic bacteria, as this ion is an essential factor for their growth and the expression of virulence factors [19]. The bactericidal effect of apo-Lf is primarily attributable to interactions with LPS, porins, and other OM proteins in Gram-negative bacteria [20–22].

Therefore, in this study we evaluated the effect of apo-BLf on the production of OMVs released by *M. haemolytica* A2, as well as on the secretion and cytotoxicity of free and OMV-harbored Lkt. The results of this study shows the potential for the use of apo-BLf in the prevention and treatment of ovine mannheimiosis.

## Materials and methods

### Lactoferrin, bacterial strain and OMV purification

Bovine apo-Lf with a purity of 97% was purchased from NutriScience Innovations, LLC, CT, USA. Apo-BLf was saturated with iron to obtain holo-BLf according to the method described by Xiao and Kisaalita [23]; iron in holo-BLf was 93% and it was quantified by an enzymatic automated method (MicroTech Laboratories, Mexico). The strain of *M. haemolytica* A2 was obtained from the pneumonic lungs of a sheep that died from mannheimiosis, and the capsular serotype was determined by indirect haemagglutination using a reference anti-serum [11]. Bacteria were grown on blood-agar plates for 24 h at 37 °C, and then an inoculum was cultured in brain heart infusion (BHI) broth for 24 h. Bacteria were harvested by centrifugation (9000 × *g* for 15 min). Culture supernatants (CS) were filtered through a 0.45 pore-size membrane (Millipore, Ireland) to remove residual cells. Finally, OMVs were recovered by ultracentrifugation (150 000 × *g* for 3 h at 4 °C) as previously described [11]. To verify that whole bacteria were not present in the OMV samples, thirty fields of OMVs were observed under an electron microscope. In addition, cultures from OMV suspensions were made in blood agar to confirm the absence of bacteria.

### Effect of apo-BLf and holo-BLf on *M. haemolytica* A2 growth

To explore the microbicidal effect of apo-BLf (iron-free) and holo-BLf (iron-saturated) in *M. haemolytica* A2, each BLf was added to 5 mL of BHI medium (total concentration of BLf: 2, 8, 16 or 20 μM). The assay was started with 10^6^ CFU, and the cells were incubated at 37 °C with agitation (180 rpm) for 3, 6 and 9 h. Cell growth was monitored by measuring the OD_595_ (Coleman Jr. II spectrophotometer, IL, USA), with each sample simultaneously plated on BHI-agar and incubated for 24 h for subsequent CFU enumeration. The experiment was performed five times, each in triplicate.

### Determination of the number of *M. haemolytica* A2 OMVs by flow cytometry

To quantify the number of OMVs, the method reported by Hernandez et al. [24] for detecting vesicles in peripheral blood from breast cancer patients was adapted for use with bacterial cell OMVs. Twenty microliters of PBS (filtered with a nitrocellulose membrane of 0.22 μm in diameter) was added to a Trucount tube (BD Biosciences, CA, USA) to hydrate the tube pearls. Then, OMVs (50 μL) were added, after which the mixture was vortexed for 30 s and then incubated for 15 min at room temperature (RT). Subsequently, PBS (450 μL) was added, after which the tube was vortexed for 30 s and then incubated for 15 min. Finally, the samples were analyzed on a flow cytometer (BD LSRFortessa, NJ, USA), and the size and granularity of the OMVs were determined. To determine the number of OMVs present in each sample, the following formula was used to analyze different populations in the dot plot: (population pearls events/population vesicles events) × (pearls per tube/sample volume). The results were analyzed using Attune^®^ Cytometric Software 2.1.0, CA, USA. Each experiment was performed three times, each in triplicate.

### Electron microscopy of whole bacteria and OMVs

Samples of bacteria grown in different sublethal concentrations of apo-BLf (2, 4, 6 and 8 µM) and in the same concentrations of holo-Lf were obtained. In addition, OMVs obtained from all cultures were placed on carbon- and Formvar-coated copper grids (Electron Microscopy Sciences, PA, USA), negatively stained via successive incubations with 2% uranyl acetate for 1 s, 30 s and 2 min, removing the uranyl after each step, and then observed under a transmission electron microscope (JEOL, JEM 2000 EX, PA, USA).

### Determination of outer membrane permeability

To determine whether the function of the bacterial OM was damaged after treatment with apo- or holo-BLf, an MIC assay was performed. This assay was carried out in 96-well plates with bacteria grown in BHI medium supplemented with different sublethal concentrations of apo- or the same concentrations of holo-BLf (1, 2, 4, 6 and 8 μM) for 2 h without agitation. Subsequently, SDS (3, 6, 9 and 15 µg/µL) or polymyxin B (3, 6, 9 and 12 μg/mL) was added and incubated for 6 h at 37 °C. Finally, the OD_595_ values and the MICs were measured (a minor MIC corresponded to a major damage of the OM) [25]. Each experiment was performed three times, each in triplicate.

### Determination of LPS in culture supernatants

Culture supernatants proteins and LPS were separated by 12% SDS-PAGE and then stained for LPS using a modified specific silver staining method [26]. Briefly, proteins were fixed in the gel overnight with a solution containing 25% isopropanol and 7% acetic acid (setting solution). Subsequently, the solution was decanted, and the gel was oxidized for 5 min in a solution containing 150 mL of deionized water, 1.05 g of periodic acid and 4 mL of setting solution. The gel was then washed eight times (20 min each) in deionized water and then stained for 10 min with a solution containing 28 mL of 0.1 N NaOH, 1.25 mL of 29.4% NH_4_OH and 5 mL of 20% AgNO_3_. Subsequently, the gel was washed four times (10 min each) in deionized water and then incubated at RT for 3–5 min in a solution containing 50 mg of citric acid in 0.5 mL of 37% formaldehyde. To preserve the gel, it was submerged for 60 min in 7% acetic acid. Subsequently, the stained gel was scanned and analyzed by densitometry using ImageJ Software (Fiji 1.51w, NIH, NY, USA).

### Detection of Lkt from *M. haemolytica* A2 in culture supernatants and OMVs by Western blot

To assess the presence of Lkt in the CS, *M. haemolytica* A2 cells (5 × 10^6^ CFU) were cultured in BHI supplemented with apo- or holo-BLf (2, 4, 6 or 8 µM, final concentration). Subsequently, the cultures were incubated for 4 h at 37 °C with agitation. Next, the cultures were centrifuged (6400 × *g*), the bacteria were discarded, and the CS was filtered through a cellulose membrane (0.22 µm diameter, Millipore). Samples from each CS were precipitated with ethanol, dried, and diluted in PBS. Protein samples (10 µL) were resolved by 8% SDS-PAGE for 2 h. Subsequently, the proteins were transferred to a nitrocellulose membrane (BioRad, Germany) at 400 mA for 1 h, after which the membrane was blocked with 0.1% TBS (Tris-buffered saline)-Tween plus 5% skim milk for 2 h and then washed in 0.1% TBS-Tween three times for 10 min each. The membrane was then incubated with a rabbit anti-Lkt Ab (Biorbyt, SF, USA) in 0.1% TBS-Tween (1:5000), washed with 0.1% TBS-Tween, incubated with a secondary peroxidase-coupled anti-rabbit Ab (1:5000) and detected by chemiluminescence.

To determine the presence of Lkt in OMVs, the same culturing procedure was performed with apo- or holo-BLf, but the bacterial cultures were incubated for 24 h. The OMVs were obtained as previously mentioned. Each experiment was performed three times, each in triplicate. Importantly, the results were analyzed by densitometry using ImageJ (Fiji) and normalized to the amount of Lkt secreted by 1 × 10^6^ bacteria for CS and Lkt harbored by 1000 vesicles for the OMV samples.

### Interaction of Lkt with ovine macrophages

#### Enrichment of ovine peripheral blood mononuclear cells (PBMCs) and their differentiation into macrophages

To obtain sheep monocyte-derived macrophages, we used a method reported for human monocytes, as we did not identify a specific method for sheep in the literature. We optimized the centrifugation step for the Lymphoprep (Axis-Shield, Norway) gradient at 1000 × *g* for 15 min (instead of 800×*g* for 20 min), because the density of sheep blood is different from that of human blood. Ovine cells were subjected to a second purification step by a magnetic separation system using human MiniMac CD14 (Miltenyi Biotec, CA, USA), following the manufacturer’s instructions. The obtained monocytes were incubated in 96-well plates in RPMI 1640 medium supplemented with 10% fetal bovine serum (FBS) (Gibco, NY, USA), 1% antibiotics and an anti-mycotic solution (Caisson Labs, UT, USA) in a CO_2_ incubator (Thermo Scientific Forma I, OH, USA) at 37 °C for 24 h to promote cell adhesion to the wells. Subsequently, the medium was changed, and 100 ng of recombinant human granulocyte macrophage-colony stimulating factor (GM-CSF) (PeproTech, Inc, NJ, USA) was added to 1 × 10^5^ cells. After 5 days, fresh GM-CSF was added every other day to maintain stimulated cell differentiation.

#### Assay of macrophage viability

Lkt secreted into the CS or OMVs released from *M. haemolytica* A2 cultured with different concentrations of apo- or holo-BLf was purified and added to cells and incubated for 1 h. In the other group, the same concentration of apo- and holo-BLf (2, 4, 6 and 8 µM) used to grow bacteria was added to RPMI medium supplemented with purified Lkt (only in the purified Lkt assay). Lkt and OMVs from bacteria grown in medium without BLf, human macrophages (Lkt assay) and cells without Lkt or OMVs were used as controls. Due to the results obtained in the pure Lkt assay, only the lowest and highest concentrations of BLf were used for the OMV experiment. After the incubation period, the medium was changed to fresh medium without phenol red and 10% FBS plus 10 µL of MTT in a final volume of 110 μL. Then, the cells were incubated for 3 h, after which DMSO was added and the absorbance of the samples was read in a spectrophotometer at 570 nm.

### Statistical analysis

Statistical comparisons among groups were performed using one-way analysis of variance (ANOVA), followed by Tukey’s posttest. Significance for all analyses was established with *P* < 0.05. All statistical analyses were performed using GraphPad Prism 6.01 (GraphPad, CA, USA).

## Results

### Apo- but not holo-BLf has bactericidal activity toward *M. haemolytica* A2

**Figure 1:**
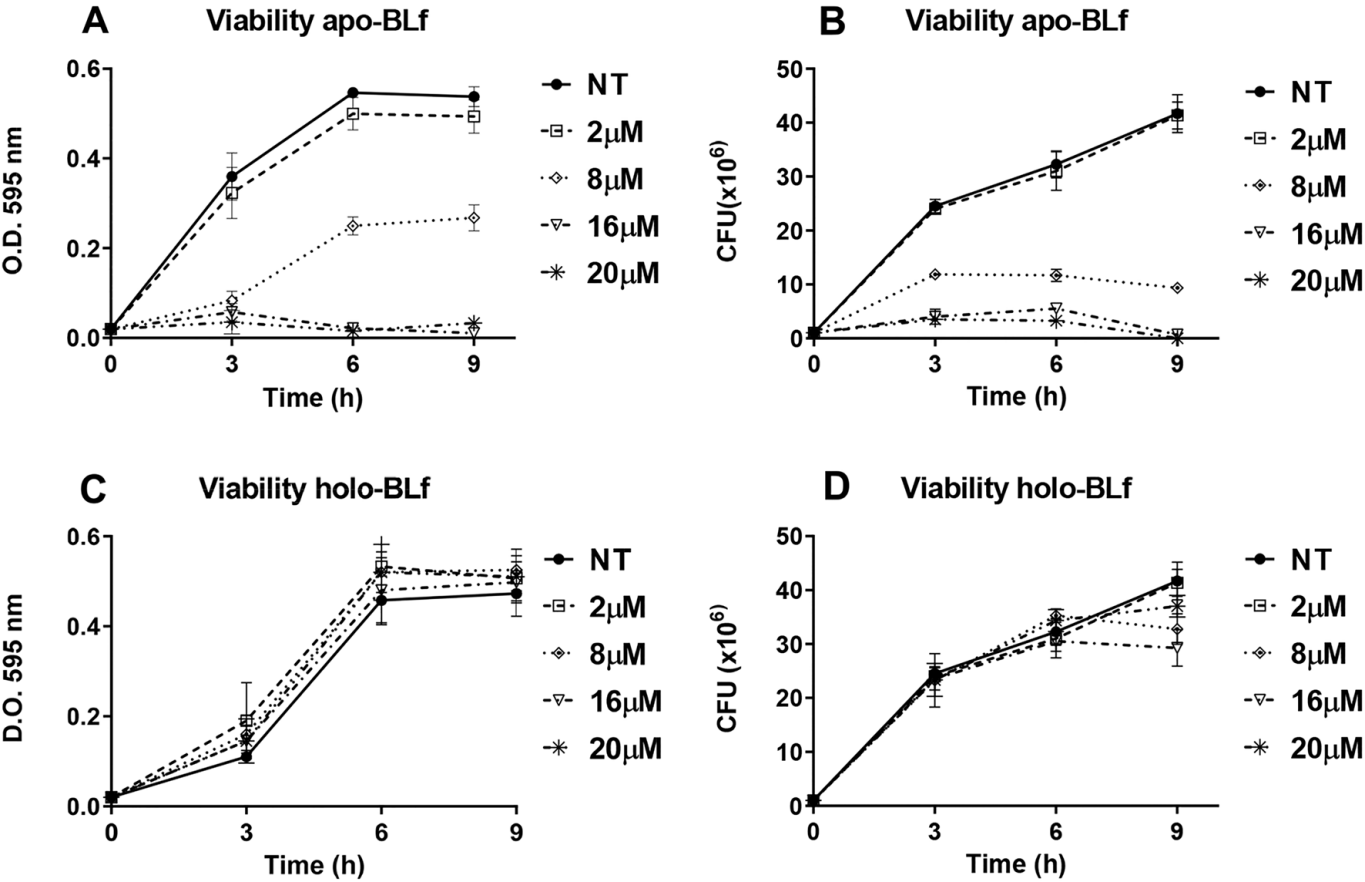
**Apo-BLf is bactericidal toward*M. haemolytica*A2, while holo-BLf does not affect the growth of this bacterium. A**, **C** OD_595_ values for the apo-BLf- and holo-BLf-treated cells, respectively. **B**, **D** Enumeration of viable bacteria for the same treatments. Bacteria were grown in BHI supplemented with different concentrations of apo- or holo-BLf.

First, apo-BLf was assessed for bactericidal activity toward *M. haemolytica* A2 using different apo-BLf concentrations and incubation times. Figure 1A shows the bacterial growth results in the presence of apo-BLf, as determined by measuring the OD_595_ values of cultures, while Figure 1B shows the viable count under the same culture conditions. Using both approaches, we observed that *M. haemolytica* was unable to grow at 16 µM apo-BLf. Apo-BLf may promote *M. haemolytica* cell death, in addition to sequestering iron, since the number of viable bacteria was drastically reduced after the third hour. This killing effect was observed after the first hour of incubation with apo-BLf (data not shown). The iron-saturated BLf form (holo-BLf) had no effect on *M. haemolytica* growth (Figures 1C and D). Interestingly, in more than 20 experiments, we observed a slight stimulation (approximately 10%) in *M. haemolytica* growth at high holo-BLf concentrations (above 10 µM), despite *M. haemolytica* being unable to utilize holo-BLf as a sole iron source [20].

### Apo-BLf increases the number of OMVs released

**Figure 2:**
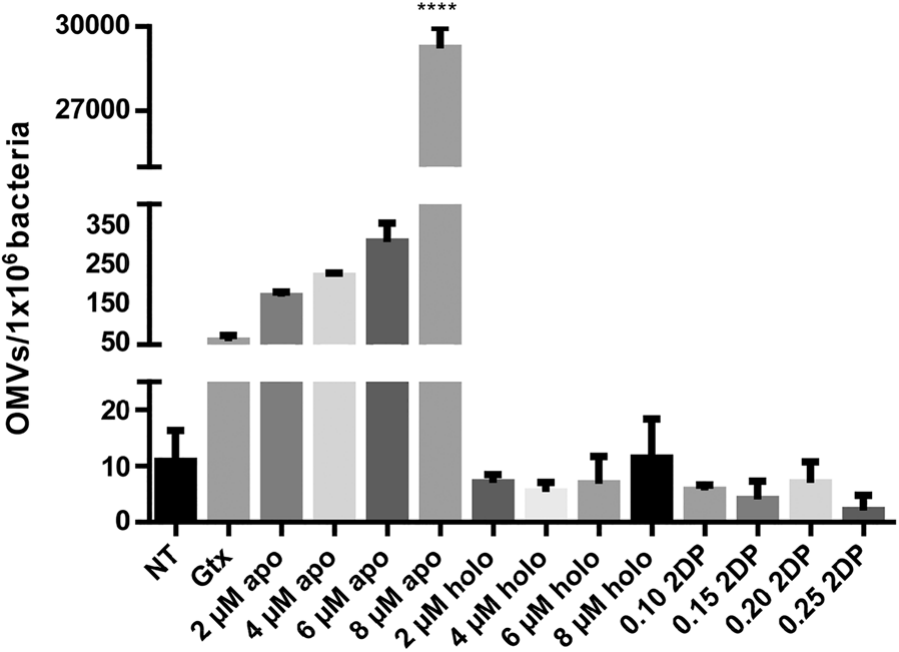
**Apo-BLf increases the release of OMVs from*M. haemolytica*A2.** Holo-BLf and 2′2-dipyridyl do not affect the number of OMVs. Bacteria were grown in BHI supplemented with 25 µg/mL gentamicin (positive control) or different concentrations of 2′2-dipyridyl, apo- or holo-BLf, after which the outer membrane vesicles (OMVs) were purified and quantified by flow cytometry. NT, no treatment; Gtx, gentamicin; apo, apo-BLf; holo, holo-BLf; 2DP, 2′2-dipyridyl. ****P < 0.0001.

As is the case for all Gram-negative bacteria, *M. haemolytica* typically produces OMVs. To assess the effect of apo-BLf on the production of OMVs, *M. haemolytica* was grown in medium supplemented with different concentrations of the glycoprotein, and the number of OMVs was quantified by flow cytometry. Since a concentration of 16 µM apo-BLf resulted in a strong decrease in the number of bacteria, only sublethal concentrations (2, 4, 6 and 8 µM apo-BLf) were used in this experiment. As gentamicin treatment increases the release of OMVs from *M. haemolytica* [11, 27], the addition of this aminoglycoside was used as a positive control. Figure 2 shows that apo-BLf increased the release of OMVs in a concentration-dependent manner. To determine whether the increase in OMV release caused by apo-BLf was due to iron chelation, we performed a similar experiment with holo-BLf (with the same concentrations used for apo-BLf) and an iron-chelating compound (2′2-dipyridyl) at different concentrations (0.10, 0.15, 0.20, 0.25 and 0.30 mM). Figure 2 shows that the number of OMVs released was not altered when holo-BLf or 2′2-dipyridyl was used at any concentration. The results of this assay suggest that apo-BLf induces the release of OMVs in an iron chelation-independent manner.

### Apo- but not holo-BLf affects the structure of both the *M. haemolytica* A2 OM and OMVs

**Figure 3:**
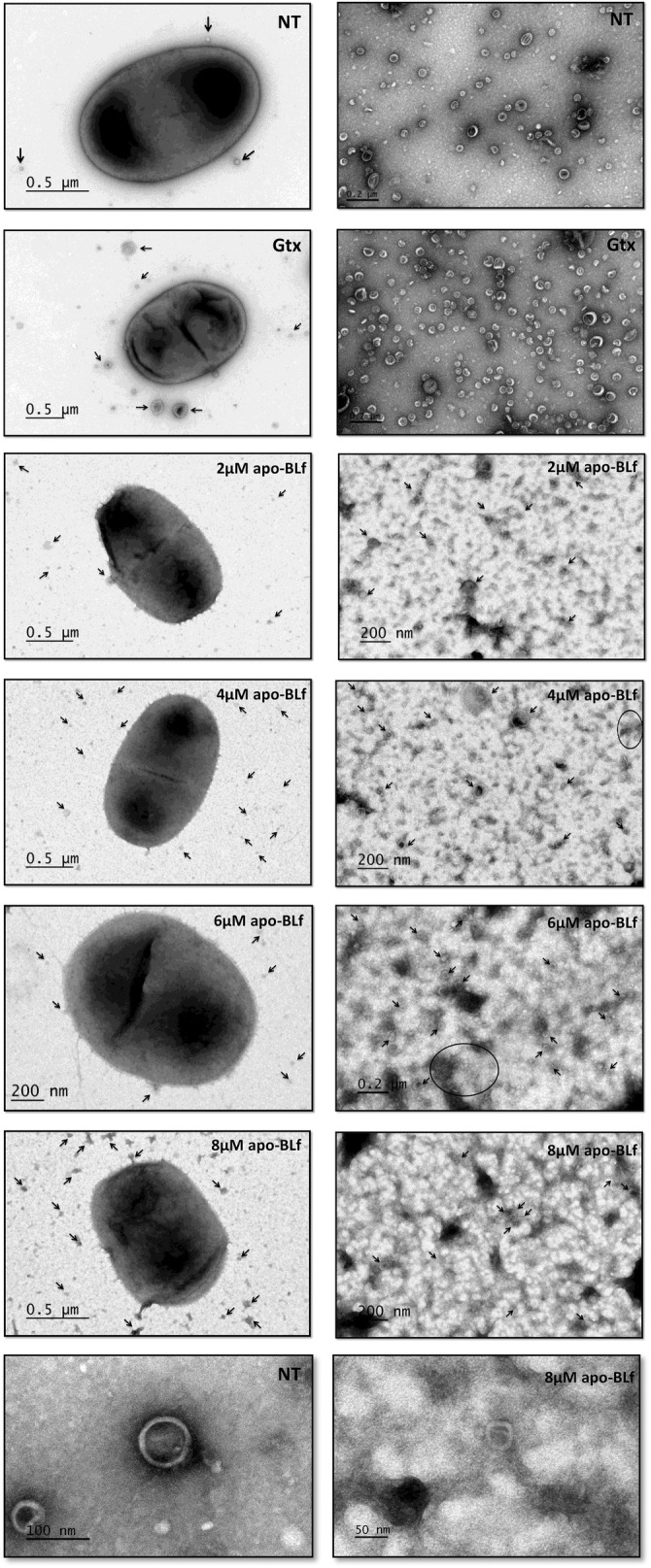
**Apo-BLf affects the OM and OMV structure of*M. haemolytica*A2.** Bacteria (left side) and purified OMVs (right side); bacteria were grown in BHI supplemented with different concentrations of apo-BLf (2, 4, 6 and 8 µM). Arrows, OMVs; circles and ovals, set of OMVs. Electron transmission microscopy, negative staining.

We subsequently investigated whether the increase in the release of OMVs from *M. haemolytica* A2 in the presence of apo-BLf could be due to apo-BLf binding to the OM, resulting in a morphological change. To this end, we analyzed the structure of the OM and OMVs produced by *M. haemolytica* A2 by negative electron microscopy. Figure 3 shows images of bacteria grown in medium supplemented with different sublethal concentrations of apo-BLf (left side) and corresponding samples of their purified OMVs (right side). In all of the groups treated with apo-BLf, the bacterial OM was affected and exhibited protrusions along the OM that presented discontinuity in some zones and generally occurred at high apo-BLf concentrations. In addition, the OMVs exhibited morphological changes, had membranes that could not be clearly observed in approximately 80% of the OMV population, and had electron-dense contents with minimal electron density (Figure 3, right side).

**Figure 4:**
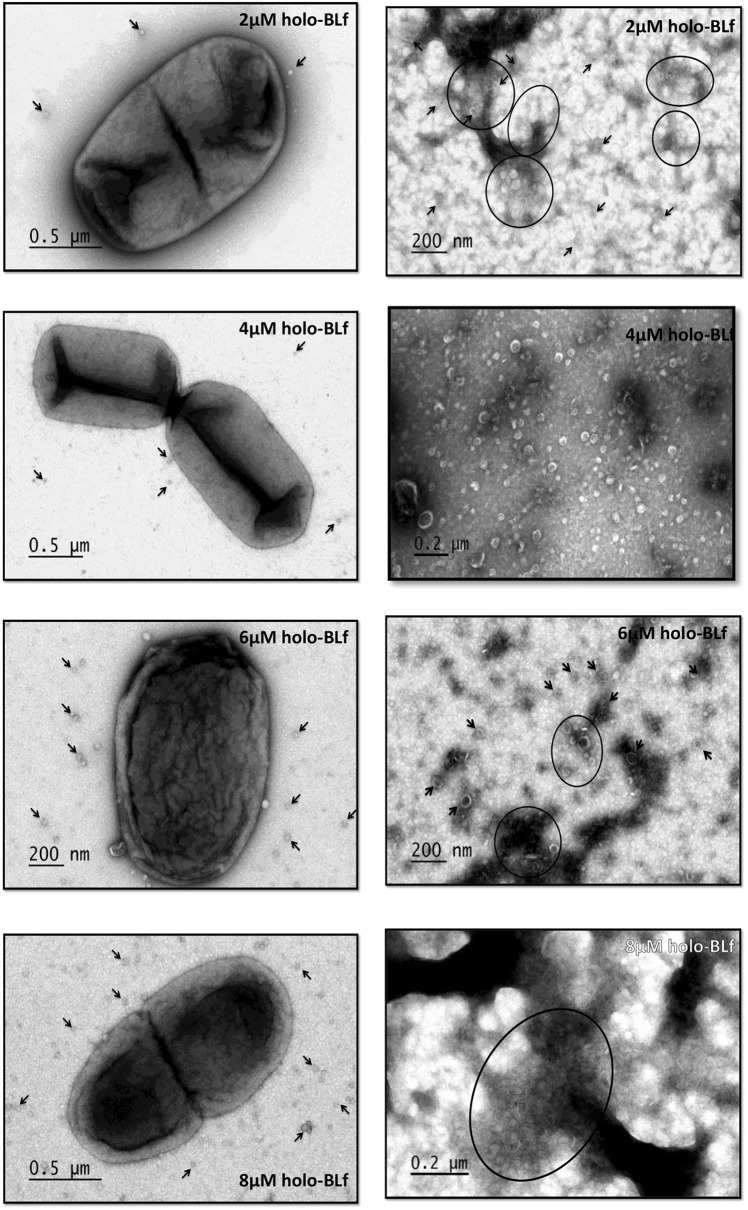
**Holo-BLf does not affect the OM and OMV structure of*M. haemolytica*A2.** Bacteria (left side) and purified OMVs (right side) were grown in BHI supplemented with different concentrations of holo-BLf (2, 4, 6 and 8 µM). Arrows, OMVs; circles and ovals, a set of OMVs. Electron transmission microscopy, negative staining.

Since the *M. haemolytica* A1 OMPs that bind to apo-BLf also bind holo-BLf [20], we assessed whether holo-BLf has the same effect as apo-BLf on the OM and OMV structure of *M. haemolytica* A2. Structural damage of the bacterial cells was not observed with holo-BLf (Figure 4, left side). However, a few number of bacteria with the abovementioned morphological characteristics detected when apo-BLf was added, were also observed (approximately 10% of the total bacteria, data not shown). In addition, the morphology of OMVs purified from *M. haemolytica* A2 treated with holo-BLf was similar to that of the control without BLf, but a decrease in the size of the OMVs was observed.

### Apo-BLf affects the permeability of the *M. haemolytica* A2 OM

**Figure 5:**
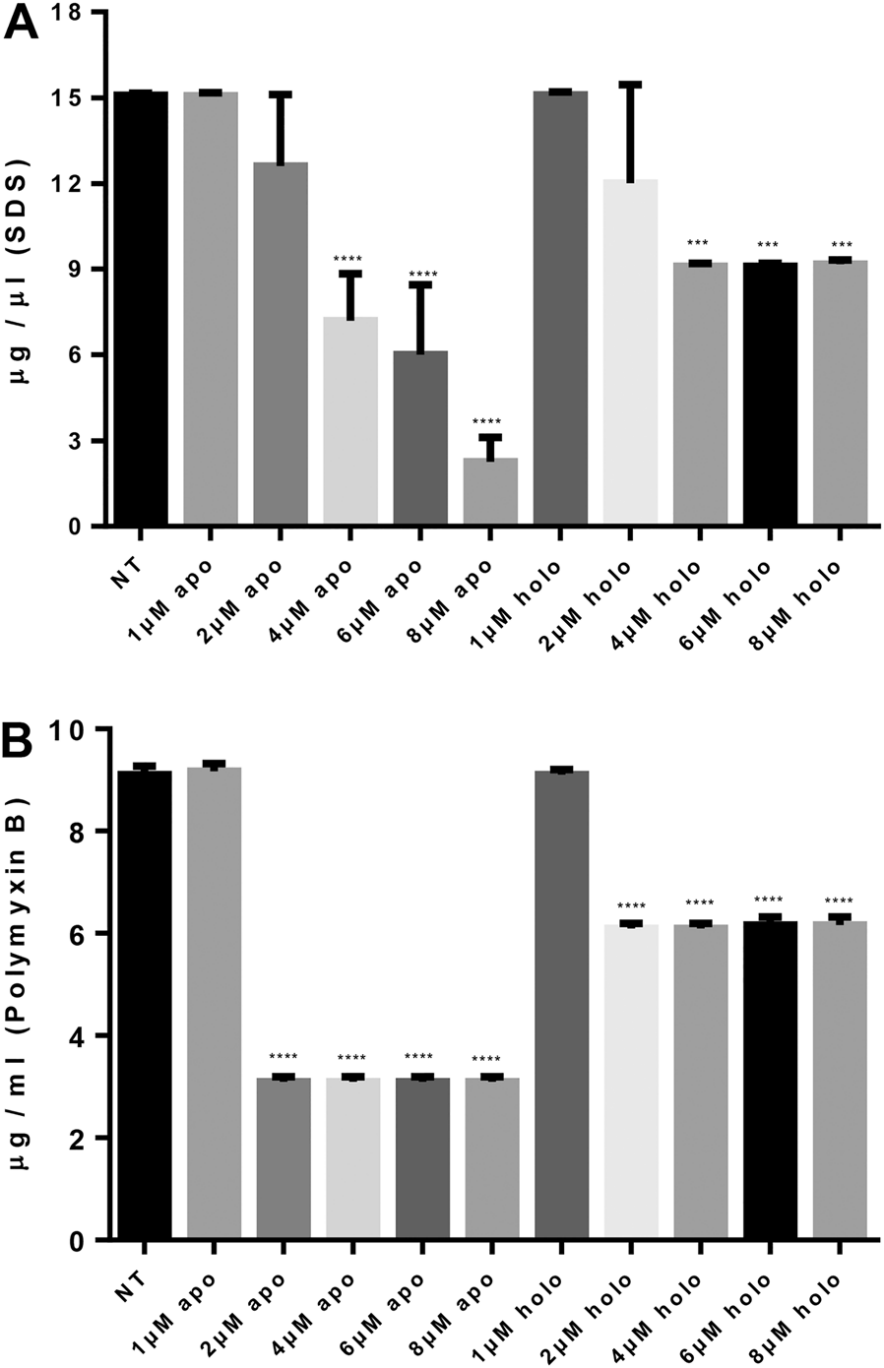
**Apo-BLf affects the outer membrane permeability of*M. haemolytica*A2.** Apo-BLf diminishes the MIC for SDS (**A**) and polymyxin B (**B**). NT, No treatment with BLf (only BHI); apo, BHI plus apo-BLf; holo, BHI plus holo-BLf. The MICs of the SDS and polymyxin B controls without BLf were 15 and 9 µg/mL, respectively. ***P < 0.001, ****P < 0.0001.

To determine whether apo-BLf destabilizes and damages the OM of *M. haemolytica* A2, thereby altering its permeability, bacterial membrane integrity assays were performed. Figure 5 shows the MIC results for SDS (Figure 5A) and polymyxin B (Figure 5B) treatments of *M. haemolytica* A2 previously grown for 2 h in BHI supplemented with apo-BLf. Bacteria grown in medium supplemented with apo-BLf showed a lower MIC toward SDS and polymyxin B than that observed for the control group, indicating that their OM was more susceptible to the entry of these compounds. This same assay was performed with holo-BLf, and a decrease in the MIC was observed, although to a much lesser degree. These results demonstrate that apo-BLf affects the permeability and continuity of the *M. haemolytica* A2 OM, which likely allows for the increased release of OMVs.

### Apo-BLf can remove LPS from the OM of *M. haemolytica* A2

**Figure 6:**
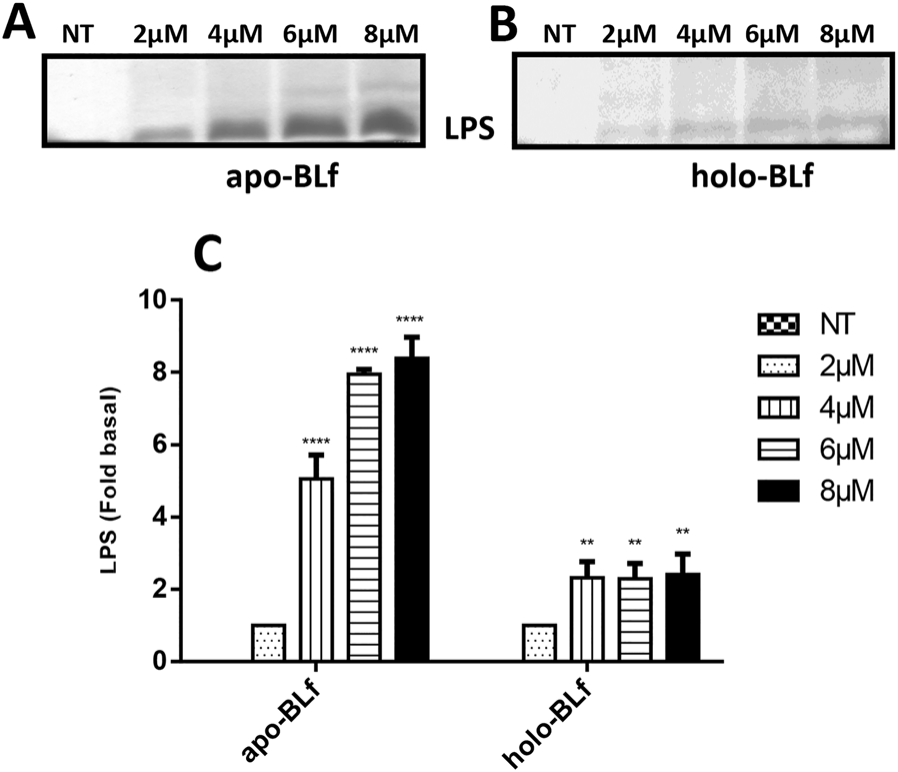
**Apo-BLf induces the release of LPS from the outer membrane of*M. haemolytica*A2.** Silver staining of LPS in SDS-PAGE gels from samples of culture supernatants of *M. haemolytica* A2 grown for 4 h in BHI supplemented with apo-BLf (**A**) or holo-BLf (**B**). **C** Graph of the LPS release data (densitometry). **P < 0.01, ****P < 0.0001.

It has been reported that the LPS embedded in the OM of Gram-negative bacteria is a Lf-binding molecule [21, 28–30], which neutralizes LPS and promotes its release while simultaneously destabilizing and permeabilizing the bacterial OM [31–34]. Thus, silver staining specific for LPS was performed for the CS from *M. haemolytica* A2 grown in BHI supplemented with different concentrations of apo-BLf. Figure 6A shows that when apo-BLf was added (at a minimum concentration of 2 µM), an LPS band was detected in the CS, and this LPS release occurred in an apo-BLf concentration-dependent manner. The densitometry graph of the LPS band is shown in Figure 6C. Next, the same experiment was performed using holo-BLf, and the results are shown in Figure 6B (silver staining) and C (densitometry). It can be clearly observed that small amounts of LPS were present in the CS when holo-BLf was used at all concentrations tested. This result is similar to that observed for the OM integrity, where apo-BLf affected the permeability of the OM to a greater extent than holo-BLf.

### Apo-BLf increases the secretion of Lkt into the CS of *M. haemolytica* A2 as a result of iron chelation

**Figure 7:**
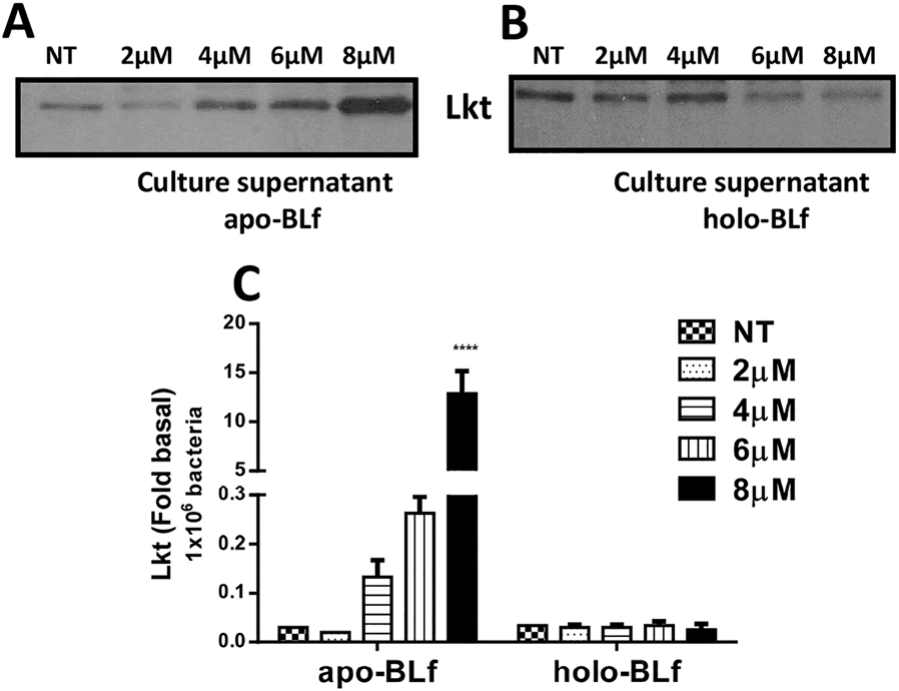
**Apo-BLf increases the secretion of leukotoxin (Lkt) into culture supernatants (CS) of*M. haemolytica*A2. A**, **B** Western blotting of Lkt secreted into CS from bacteria grown in BHI supplemented with apo- or holo-BLf, respectively. **C** Graph of Lkt secreted into CS (densitometry, adjusted to Lkt release by 1 × 10^6^ bacteria). ****P < 0.0001.

We evaluated the secretion of Lkt into the CS by *M. haemolytica* A2 grown under different treatments. Figure 7A shows the Western blot results for the CS samples of bacteria grown in medium supplemented with apo-BLf, where the increase in Lkt secretion was dependent on its concentration such that the greatest effect was observed at 8 µM apo-BLf. Additionally, to evaluate whether the increase in Lkt secretion was due to the iron-chelating ability of apo-BLf, we performed this assay with holo-BLf (Figure 7B) and 2′2-dipyridyl (Additional file 1). In contrast to the results observed using apo-BLf, the samples treated with holo-BLf did not show an increase in Lkt secretion. However, for the 2′2-dipyridyl-treated samples, we observed an increase in Lkt secretion, which was more evident at concentrations of 0.20 and 0.25 mM, similar to apo-BLf. Thus, we suggest that the observed increase in Lkt secretion into the CS could be partially due to the iron-chelation activity of apo-BLf.

**Figure 8:**
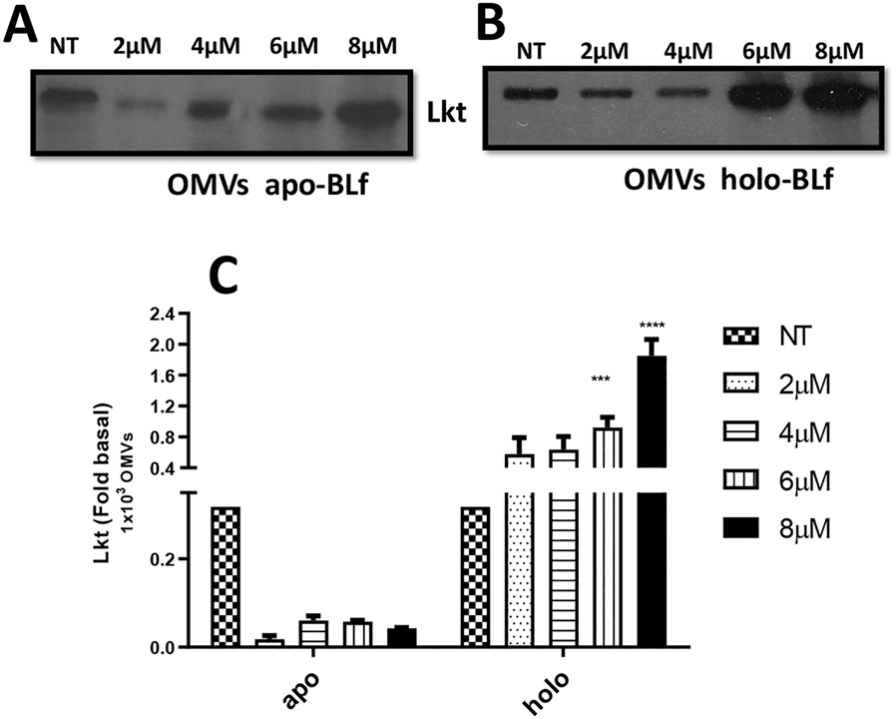
**Holo-BLf increases the leukotoxin (Lkt) concentration in OMVs released by*M. haemolytica*A2. A**, **B** Western blotting of Lkt contained within OMVs of bacteria grown in BHI supplemented with apo- or holo-BLf, respectively. **C** Graph of Lkt contained within OMVs (densitometry, adjusted to Lkt release from 1000 OMVs). ***P < 0.001, ****P < 0.0001.

The Lkt content in OMVs was also measured and showed different results compared to those observed for the CS samples. Although an increase in Lkt was observed in the samples with high concentrations of apo-BLf, when these values were adjusted for the number of vesicles released, an increase in the Lkt contents of OMVs was not observed (Figures 8A and C). The same increase in Lkt was observed in the holo-BLf-treated samples (Figures 8B and C), but unlike the apo-BLf-treated samples, this increase was maintained when adjusted for the number of vesicles released, suggesting that the holo-BLf treatment increased the secretion of Lkt contained in OMVs.

### Lkt from the CS and OMVs of *M. haemolytica* A2 grown in medium supplemented with apo- and holo-BLf are cytotoxic toward ovine macrophages

**Figure 9:**
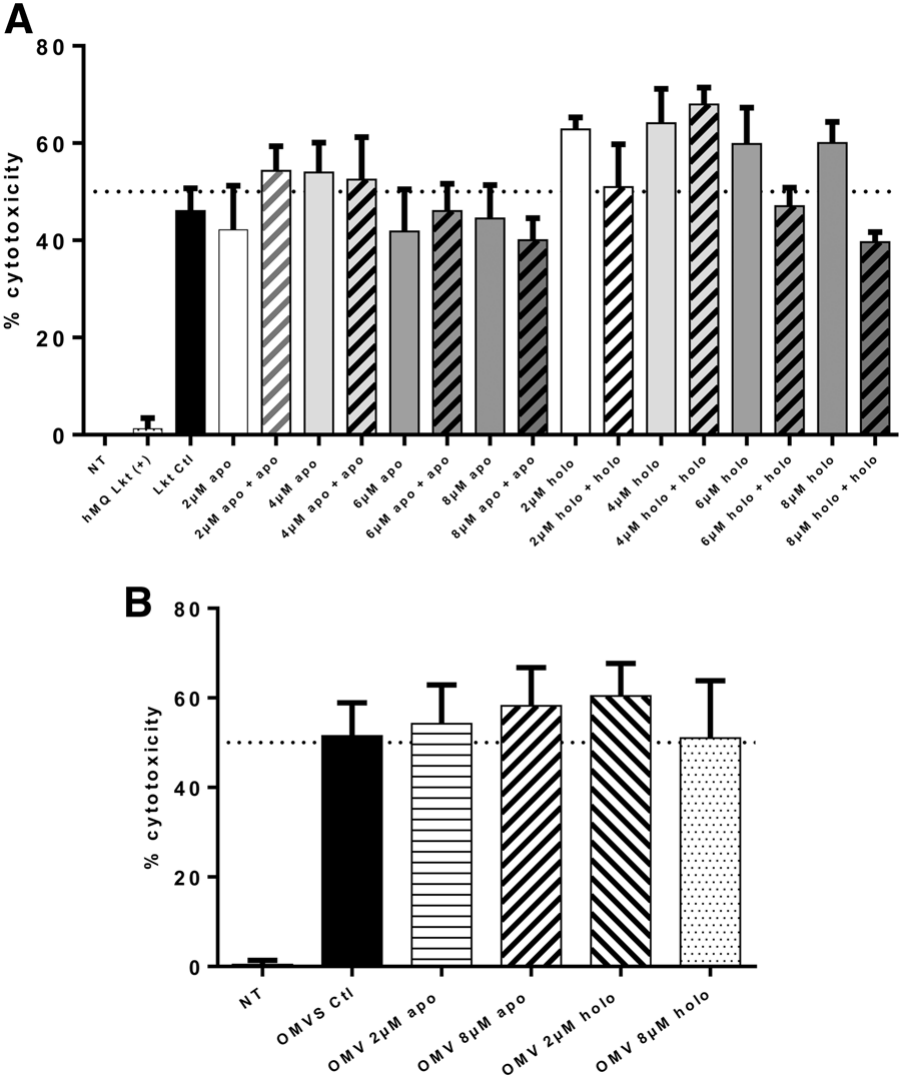
**Lkt from*M. haemolytica*A2 grown in medium supplemented with apo- and holo-BLf is active. A** Ovine macrophages were treated with purified Lkt secreted by *M. haemolytica* A2 grown in medium supplemented with apo- and holo-BLf (2, 4, 6 and 8 µM) or purified Lkt plus RPMI with the same concentration of apo- and holo-Lf (2, 4, 6 and 8 apo- plus apo-Lf or 2, 4, 6 and 8 holo- plus holo-Lf). **B** Ovine macrophages were treated with OMVs released by *M. haemolytica* A2 grown in medium supplemented with apo- and holo-BLf (2 or 8 µM).

An MTT assay was performed on ovine macrophages by treating cells with purified Lkt secreted by *M. haemolytica* A2 grown in the presence of apo- or holo-BLf (bacteria grown in BHI were used as a control), after which the Lkt cytotoxicity was measured. Lkt had a toxic effect toward ovine macrophages in all groups analyzed, and the same result was obtained when apo- or holo-BLf was added to the cell medium (Figure 9A). Human macrophages were used as a control group, where the Lkt of *M. haemolytica* had no toxic effect. Therefore, we concluded that the Lkt released from cells grown in medium supplemented with apo- or holo-BLf is active and presents classic cytotoxic activity. With respect to OMVs (Figure 9B), the Lkt from bacteria grown with apo-BLf and holo-BLf was also cytotoxic. These results indicate that CS and OMV Lkt is biologically functional.

## Discussion

The increase in the resistance of *M. haemolytica* to antibiotics worldwide has created an urgent need for the investigation of novel alternative strategies for the treatment and prevention of ovine mannheimiosis. Lf is a multifunctional glycoprotein that has several activities, the most studied of which is as an antimicrobial compound. In this study, the effect of apo- and holo-BLf on the growth and OMV production of *M. haemolytica* A2 was evaluated. Apo-BLf showed a bactericidal effect toward *M. haemolytica* A2, a result that is in agreement with those of other reports, such as for *Streptococcus pneumoniae* [35], *Vibrio cholerae* [36], antibiotic resistant strains of *Helicobacter pylori* [37], *Staphylococcus aureus* and *Escherichia coli* [38], *Pseudomonas fluorescens* in ground beef [39], and *Actinobacillus pleuropneumoniae* [40]. In contrast, when we performed this assay using BHI supplemented with holo-BLf, we observed that this ferric glycoprotein did not inhibit *M. haemolytica* A2 growth.

Outer membrane vesicles are spherical structures that are formed from the OM. OMVs enclose a broad range of membrane-associated and periplasmic proteins, a few cytosolic components and DNA fragments, all of which have important biological functions, such as in stress responses, quorum sensing, virulence, and target host cell signaling. Many OMV components may represent an effective long-distance delivery mechanism for a broad range of effectors/virulence factors to host cells [41]. The contents and production of OMVs have been reported to be affected by the growth conditions of bacteria [41–43]. Furthermore, the aminoglycoside antibiotic gentamicin can also perturb the packing order of lipids, thereby destabilizing the bilayer membrane and increasing the release of vesicles by some bacteria, such as *Pseudomonas aeruginosa* [44].

The effect of mammalian Lf on bacterial OMV release has not been explored. Therefore, we first evaluated the number of OMVs released by *M. haemolytica* when grown in medium supplemented with different sublethal concentrations of apo- and holo-BLf. An increase in the release of OMVs with apo-BLf but not with holo-BLf was detected. Since apo-BLf is an iron-chelating protein, 2′2-dipyridyl was used as a control for iron starvation conditions. As with holo-BLf, there was no change in the number of OMVs when bacteria were treated with 2′2-dipyridyl. Similar results were reported by Chan et al. [41], who observed that iron-limiting conditions had a minimal effect on the number of OMVs produced from clinical isolates of extraintestinal pathogenic *E. coli* (ExPEC). Thus, we suggest that the lack of iron in the growth medium does not induce the exacerbated release of OMVs and that the apo-BLf-mediated increase in the release of vesicles is due to another mechanism of action, such as the binding of apo-BLf to OM components, which produces membrane modifications that alters its functions.

Multiple reports have described the damage caused by Lf to the OM of different bacterial species, which has also been observed to be caused by Lf N-terminus-derived peptides named lactoferricins (Lfcins) [31–33, 35]. Thus, we assessed whether the increase in the apo-BLf-mediated increase in OMV production is due to the effect on the structure and function of the OM. We observed severe OM damage for all apo-BLf concentrations assayed and a clear increase in the number of released OMVs with an altered morphology. Similar results were obtained using the Lfcin peptide from BLf by Yamauchi et al. [33], who showed that *E. coli* O157:H7 exposed to BLfcin (100 µg/mL) showed an altered cell membrane morphology that included the appearance of membrane “blisters”, although these structures were not studied. León-Sicairos et al. [35] reported that *Streptococcus pneumoniae* (a Gram-positive bacterium) cells treated with BLfcin (40 µM) displayed deformation and thickening of the cell wall as well as thickened septa with irregular features when treated with a BLf chimera [a fusion peptide between Lfcin and lactoferrampin (Lfampin), a peptide containing the 265–284 amino acid region], where atypical bubbling and increased permeability of the inner membrane were observed. In *E. coli* K12, Lfcin and Lfampin (20 µM each) also lead to OM breakage in such a way that the inner and OM fused and protrusions from the surface were observed. In this case, the authors reported vesicle-like structures of approximately 50 nm in diameter in more than 50% of the cells [45]. Taken together, these results indicate that apo-Lf and its derivate peptides damage bacterial membranes and affect their functionality. Regarding the effects of iron saturation of Lf, the results of electrophoretic and crystallographic studies have indicated that iron chelation of this molecule significantly alters the structural conformation of the protein, as holo-Lf has a more closed structure and is more resistant to high temperature and pH values than apo-Lf [46–48]. Such a conformational change may influence its interaction with bacterial membranes. In our assays with holo-BLf, damage to OM and OMV structures was not observed. Therefore, we suggest that due to this conformational change, holo-BLf protein is unable to affect the bacterial OM, although this iron-loaded protein is able to bind to it.

The OM has an asymmetric lipid bilayer with negatively charged LPS molecules that are primarily localized on the outer leaflet and are stabilized by the presence of divalent cations [49]. Multiple observations have shown that Lf causes LPS release, suggesting that Lf has membrane-permeabilizing activity [31–33, 35]. We assessed the release of LPS from *M. haemolytica* A2 grown in medium supplemented with apo- and holo-BLf. Apo-BLf-mediated LPS release occurred in a concentration-dependent manner, and holo-BLf also promoted LPS release, although to a much lesser degree than apo-BLf. Additionally, *M. haemolytica* grown in the presence of apo-BLf displayed diminished MIC value for SDS and polymyxin B. Apo-BLf decreased the functions of the OM, causing this structure to be more susceptible to external compounds. In contrast, holo-BLf permeabilized the OM of *M. haemolytica* to a lesser degree, a result that was consistent with our previous data and electron microscopy images. We suggest that the increase in OMVs released by *M. haemolytica* grown in medium supplemented with apo-BLf is in part due to its ability to remove divalent cations, such as Ca^2+^ and Mg^2+^ [32, 34], thereby destabilizing LPS, inducing damage to the OM and causing increasing cell permeability, which allows OMVs to be more easily released. This effect was not observed for holo-BLf, perhaps because differences in its tertiary structure does not allow for similar binding to the OM such that it cannot harm bacterial cells.

The ability of bacterial pathogens to adapt to the environment within the host is essential for their virulence [50], and environmental signals have been shown to influence virulence gene expression in several different organisms. Microorganisms have adapted to iron limitation in mammalian hosts by evolving diverse mechanisms for the assimilation of sufficient iron for growth. In some organisms, iron starvation leads to a response through an increase in the expression of RTX toxins and hemolysins, which can lyse host cells and cause them to release their internal stores of iron [51]. We demonstrated that apo-BLf, an iron-chelator, increased the secretion of Lkt into the CS. Moreover, the addition of the iron-chelator 2′2-dipyridyl also increased the secretion of Lkt, indicating that Lkt secretion partially depends on iron. Similar results were reported by Marciel and Highlander [52], who showed that in the presence of 2′2-dipyridyl, the transcription of the *M. haemolytica lkt* gene promoter was increased more than threefold. These authors performed a detailed study of the transcriptional regulation of this gene through the development of plasmid-borne chloramphenicol acetyltransferase (cat) operon fusions and concluded that the *lkt* gene is negatively regulated by iron [52]. Balashova et al. [53] also showed that the secretion of leukotoxin by *Aggregatibacter actinomycetemcomitans*, another member of the Pasteurellaceae family, is increased in low-iron medium. *M. haemolytica* is capable of reaching the lungs, where the microenvironment is low in iron. Thus, as a mechanism for *M. haemolytica* to obtain iron for growth, it seems reasonable that iron limitation leads to increased Lkt production, which destroys cells and releases iron. This mechanism has been reported in other bacterial species [51].

Other researchers [6, 54] have reported a positive effect of iron on the regulation of Lkt secretion in *M. haemolytica*. The difference between these observations and our results may be due to apo-BLf being a multifunctional protein with activities other than as a bacteriostatic agent as an iron-chelator, since it has bactericidal activity by being able to bind and damage the bacterial OM. Therefore, the apo-BLf iron-chelating effect may not be the only one involved in the regulation of Lkt secretion.

Interestingly, only holo-BLf increased the level of Lkt present in OMVs. Perhaps this result is due to its ability to promote the production of smaller vesicles than those typically produced by *M. haemolytica* A2, many of which could not be detected by flow cytometry. Thus, it is necessary to use other methods to accurately evaluate this effect, such as nanoparticle tracking analysis.

Although this study reports the basic interactions between BLf and some *M. haemolytica* A2 components (OM and LPS), as well as the effect on some virulence factors, such as Lkt and OMVs, our results can be informative for further studies of the use of apo-BLf in the treatment of ovine mannheimiosis. In the future, assays with animals could be performed to determine the appropriate apo-BLf doses for its possible use as an adjuvant in the treatment of ovine mannheimiosis and assess its synergy with antibiotics.

In summary, the results of this study demonstrates that apo-BLf is bactericidal toward *M. haemolytica* A2. At sublethal concentrations, apo-BLf promotes OMV release, which is dependent on the apo-BLf concentration, induces OM damage and affects its permeability. Apo-BLf increases Lkt secretion via an iron-chelating effect, and Lkt and OMVs released from *M. haemolytica* grown in medium supplemented with apo- or holo-BLf maintains its toxic effect toward ovine macrophages. Due to these effects, the use of apo-BLf can be recommended for the treatment of ovine mannheimiosis, but the appropriate doses must be determined.

## Supplementary information

**Additional file 1. 2′2-Dipyridyl increases leukotoxin (Lkt) secretion into*M. haemolytica*A2 culture supernatants (CS).** Bacteria were grown in BHI supplemented with 2′2-dipyridyl. **A** Western blotting of Lkt in the CS of *M. haemolytica* A2. **B** Graph of Lkt (densitometry). ****P < 0.0001.

## Abbreviations

OM: outer membrane
OMVs: outer membrane vesicles
Lf: lactoferrin
apo-BLf: apo-bovine lactoferrin
holo-BLf: holo-bovine Lactoferrin
Lkt: leukotoxin
LPS: lipopolysaccharide
OMPs: outer membrane proteins
CS: culture supernatants
BHI: brain heart infusion
CFU: colony forming units
RT: room temperature
PBS: phosphate-buffered saline
MIC: minimum inhibitory concentration
SDS: sodium dodecyl sulfate
TBS: tris-buffered saline
PBMC: peripheral blood mononuclear cells
FBS: fetal bovine serum
GM-CSF: granulocyte macrophage-colony stimulating factor
DMSO: dimethyl sulfoxide
ANOVA: one-way analysis of variance
2DP: 2′2 dipyridyl
Lfcins: lactoferricins
Lfampin: lactoferrampin
NT: no treatment
Gtx: gentamicin
