# Supplementary figures and images for: Effect of apo-lactoferrin on leukotoxin and outer membrane vesicles of Mannheimia haemolytica A2
Source: Vet Res. 2020 Mar 5;51:36. doi: 10.1186/s13567-020-00759-z (PMC7059318; doi:10.1186/s13567-020-00759-z)

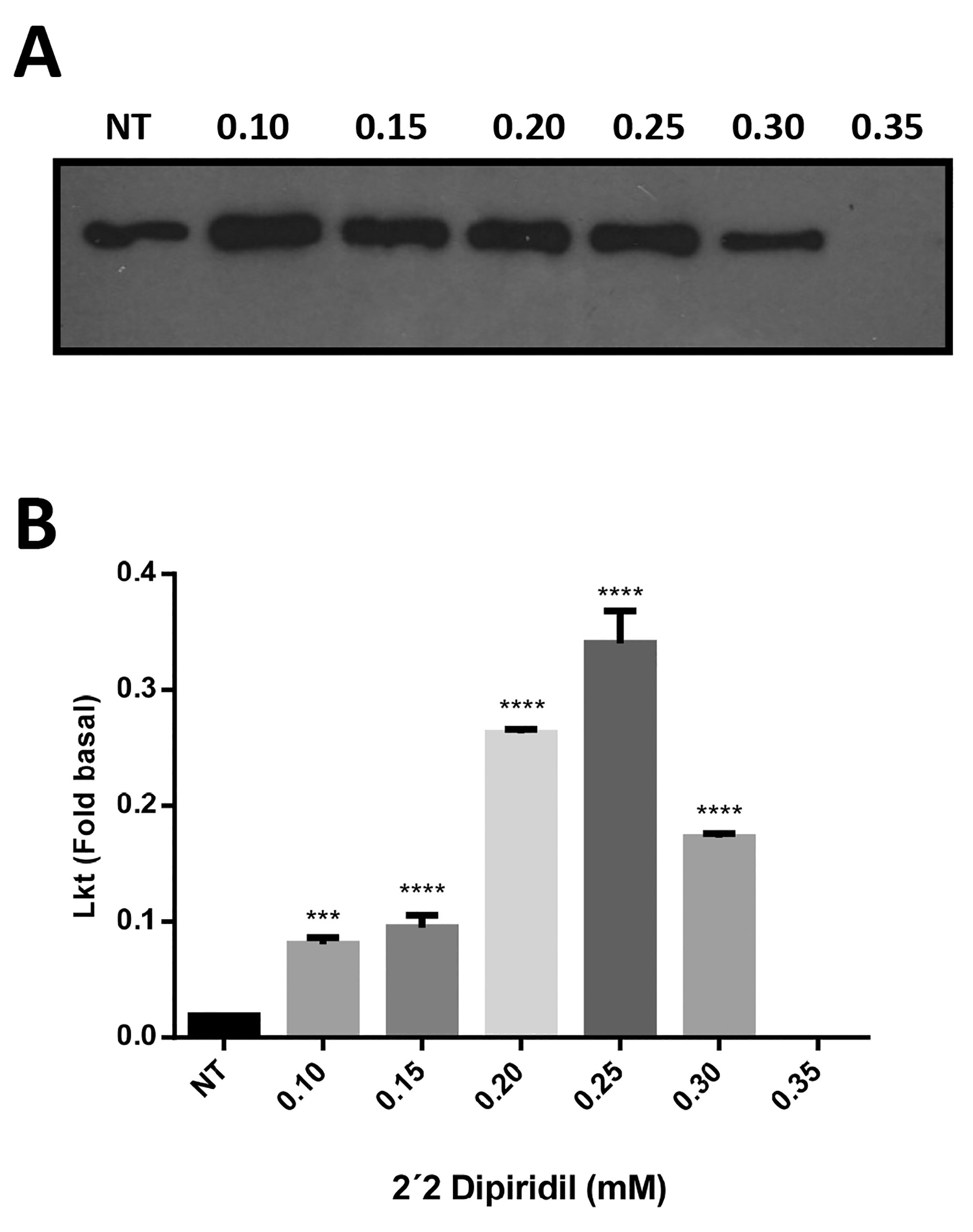

Supplement: Supplementary file 1 — Additional file 1. 2′2-Dipyridyl increases leukotoxin (Lkt) secretion intoM. haemolyticaA2 culture supernatants (CS). Bacteria were grown in BHI supplemented with 2′2-dipyridyl. A Western blotting of Lkt in the CS of M. haemolytica A2. B Graph of Lkt (densitometry). ****P < 0.0001. [file 13567_2020_759_MOESM1_ESM.tif]
